# Prenatal Nutritional Intervention Reduces Autistic-Like Behavior Rates Among *Mthfr*-Deficient Mice

**DOI:** 10.3389/fnins.2019.00383

**Published:** 2019-05-02

**Authors:** Ayelet Orenbuch, Keren Fortis, Siraphat Taesuwan, Raz Yaffe, Marie A. Caudill, Hava M. Golan

**Affiliations:** ^1^Department of Physiology and Cell Biology, Faculty of Health Sciences, Ben-Gurion University of the Negev, Beersheba, Israel; ^2^Division of Nutritional Sciences, Cornell University, Ithaca, NY, United States; ^3^Division of Food Science and Technology, Faculty of Agro-Industry, Chiang Mai University, Chiang Mai, Thailand; ^4^Zlotowski Center for Neuroscience, Ben-Gurion University of the Negev, Beersheba, Israel

**Keywords:** autism, one-carbon metabolism, choline, GABA, glutamate receptors, H3, pregnancy, postnatal

## Abstract

The causes and contributing factors of autism spectrum disorders (ASD) are poorly understood. One gene associated with increased risk for ASD is methylenetetrahydrofolate-reductase (*MTHFR*), which encodes a key enzyme in one carbon (C1) metabolism. The *MTHFR 677C > T* polymorphism reduces the efficiency of methyl group production with possible adverse downstream effects on gene expression. In this study, the effects of prenatal and/or postnatal diets enriched in C1 nutrients on ASD-like behavior were evaluated in *Mthfr*-deficient mice. Differences in intermediate pathways between the mice with and without ASD-like behaviors were tested. The findings indicate that maternal and offspring *Mthfr* deficiency increased the risk for an ASD-like phenotype in the offspring. The risk of ASD-like behavior was reduced in *Mthfr*-deficient mice supplemented with C1 nutrients prenatally. Specifically, among offspring of *Mthfr*+/- dams, prenatal diet supplementation was protective against ASD-like symptomatic behavior compared to the control diet with an odds ratio of 0.18 (CI:0.035, 0.970). Changes in major C1 metabolites, such as the ratios between betaine/choline and SAM/SAH in the cerebral-cortex, were associated with ASD-like behavior. Symptomatic mice presenting ASD-like behavior showed decreased levels of GABA pathway proteins such as GAD65/67 and VGAT and altered ratios of the glutamate receptor subunits GluR1/GluR2 in males and NR2A/NR2B in females. The altered ratios, in turn, favor receptor subunits with higher sensitivity to neuronal activity. Our study suggests that MTHFR deficiency can increase the risk of ASD-like behavior in mice and that prenatal dietary intervention focused on *MTHFR* genotypes can reduce the risk of ASD-like behavior.

## Introduction

The *MTHFR*-coding gene is associated with increased risk for developmental outcomes, including ASD and schizophrenia (James et al., 2006; Feng et al., 2009; Goin-Kochel et al., 2009; Mohammad et al., 2009; Vares et al., 2010; Liu et al., 2011; Schmidt et al., 2011; Guo et al., 2012; Schmidt et al., 2012; Pu et al., 2013). The activity of MTHFR strongly affects the one-carbon (C1) metabolic pathway, which is central to cellular methylation reactions. The C1 metabolic pathway includes the folate dependent and folate independent pathways, which converge to convert homocysteine to methionine. In the folate dependent pathway, MTHFR catalyzes the reduction of methylene-tetrahydrofolate to methyl-tetrahydrofolate, which then reacts with homocysteine. The folate independent pathway uses choline to produce betaine, which also remethylates homocysteine. Methionine produced from either pathway is converted to *S*-adenosylmethionine (SAM), a universal methyl donor. Investigations of C1 metabolism in humans with the homozygous recessive *MTHFR677TT* (vs. *677CC*) polymorphism, and thus, impaired MTHFR activity, revealed increased use of the methyl donor choline (Yan et al., 2011) or its derivative betaine (Ganz et al., 2016, 2017) to preserve methyl group homeostasis. Similar findings have been reported in *Mthfr*-knockout mice (Schwahn et al., 2003; Chew et al., 2011).

Among ASD patients and their mothers, *MTHFR677TT* polymorphism frequency is higher than that in the general population (Boris et al., 2004; James et al., 2006; Goin-Kochel et al., 2009; Mohammad et al., 2009; Liu et al., 2011; Guo et al., 2012; Schmidt et al., 2012; Pu et al., 2013). The results of a large case-control study indicate that C1 metabolism enzymes in the *in utero* environment have additive effects on normal neurogenesis (Schmidt et al., 2011). This study also suggested that the risk for ASD children among mothers with *MTHFR 677TT* was reduced when folic acid and prenatal vitamin supplements were taken periconceptionally and in the first trimester of pregnancy (Schmidt et al., 2011, 2012). The higher abundance of the polymorphic allele *MTHFR 677T* in ASD children compared to the general population was confirmed by a meta-analysis (Pu et al., 2013), and a protective effect was reported for folic acid fortification (Surén et al., 2013).

Another strategy to reduce the risk of ASD in the offspring of mothers with *MTHFR* deficiency may be to supplement maternal diet with extra choline, since neither it nor betaine require MTHFR activity for their use in methyl metabolism. Indeed, a significant subgroup of children diagnosed with ASD were found to have inadequate levels of choline and betaine, and extra choline during the postnatal period has been shown to benefit ASD children who exhibit low C1 metabolism activity (Hamlin et al., 2013). Maternal choline supplementation during pregnancy and lactation was tested recently in the BTBR mouse model of ASD, a strain that carries the polymorphic alleles of several genes related to choline and to one carbon metabolism (Langley et al., 2014). Adult BTBR offspring of choline supplemented mothers exhibited improved performance in tests measuring repetitive behavior, anxiety and sociability, indicating that choline supplementation has long-term benefits (Langley et al., 2014). Notably, supplementation of *Mthfr*-knockout mice with betaine, a choline derivative, during pregnancy and weaning also promoted survival and normal brain morphological development (Schwahn et al., 2004; Chen et al., 2005). Thus, although more evidence is needed, betaine supplementation may be another viable route for ASD treatment. In the *Mthfr* KO mouse model, we recently showed delayed morphogenesis that was governed solely by maternal genotype, an observation that emphasizes the dependency of newborns on the supply of C1 metabolites from the mother. The robust effect of maternal genotype, wherein mice behavior in tests that measure ASD core symptoms was impaired by maternal genotype in interaction with offspring genotype, was observed at pre-pubertal ages and maintained through adulthood (Sadigurschi and Golan, 2018).

Collectively, human and mouse data suggest that C1 metabolism deficiency can be compensated for by adjusting maternal and possibly newborn nutrient intakes to their specific needs as defined by their genotype. In the current study, our goal was to rescue the behavioral phenotype associated with *in utero* MTHFR deficiency using a diet enriched with C1 nutrients (folate, choline, and betaine) given during two periods of offspring development: (i) periconceptional period and pregnancy, and (ii) lactation and weaning. Additionally, the genetic and nutritional effects of the enriched diet on C1 metabolism and other potential intermediate pathways were investigated.

## Materials and Methods

### Mouse Colony

Mice with a Balb/cAnNCrlBR background and heterozygous for the *Mthfr*- knock out were studied (mice kindly provided by Prof. Rima Rozen, McGill University, Montreal, QC, Canada). The *Mthfr* gene in these mice was interrupted by the insertion of the Neo^r^ gene as described before (Chen et al., 2001). The mouse colony was maintained in the animal facility of Ben-Gurion University of the Negev on a 12:12 h light/dark schedule with food and water provided *ad libitum*. All experiments were carried out in accordance with the National Institutes of Health guidelines for the care and use of laboratory animals (NIH Publications No. 8023, revised 1978) and with the guidelines of the Israeli Council on Animal Care. The study protocol was approved by the animal care and use committee of Ben-Gurion University of the Negev.

### Experimental Design

Five groups of mice from each sex were tested, and in all cases, the paternal genotype was *Mthfr*+/+. Group 1 consisted of Wt offspring born to wild-type dams that were fed a control diet (Wt-Wt-CD). Group 2 consisted of Wt offspring born to *Mthfr*+/- dams that were fed a control diet (Wt-Het-CD). Group 3 consisted of *Mthfr*+/- offspring born to the *Mthfr*+/- dams in Group 3 and also fed the control diet (Het-Het-CD). Groups 4 and 5 were similar to Group 3, except that the diets of the former were enriched with C1 nutrients. Group 4 dams were fed with an enriched diet during both the periconceptional period (three weeks before conception) and pregnancy (Het-Het-PP). The resulting offspring were fostered at postnatal day 1 by *Mthfr*+/- dams fed with the CD. Finally, Group 5 dams were fed the control diet during pregnancy, and the offspring were fostered, beginning on postnatal day 1 and during their first month of life, by *Mthfr*+/- dams fed an enriched diet [postnatal (PN), Het-Het-PN]. Thus, lactating pups were exposed to the enriched diet via maternal nutritional status and, when they began to search for food and water in the cage, via nutritional fortification in the drinking water. To control for the effect of fostering on newborn development, the newborns from groups 1–3 were also fostered by dams of the same groups on postnatal day 1. At age 30 days, mice were separated from dams and littermates and housed together in groups of four mice of similar age and sex. Each cage contained mice from different experimental groups. The number of mice in each group is presented in Table 1.

**Table 1:** Percent of symptomatic mice, Classes 1 and 2.

Sex		Male					Female				
Group		1	2	3	4		1	2	3	4	5	
				
Genotype	Maternal	MTHFR+/+	MTHFR+/-	MTHFR+/-	MTHFR+/-		MTHFR+/+	MTHFR+/-	MTHFR+/-	MTHFR+/-	MTHFR+/-	
	Offspring	MTHFR+/+	MTHFR+/+	MTHFR+/-	MTHFR+/-		MTHFR+/+	MTHFR+/+	MTHFR+/-	MTHFR+/-	MTHFR+/-	

Diet		CD	CD	CD	PP	All	CD	CD	CD	PP	PN	All
Number of mice	All	12	13	10	11	46	13	8	12	8	7	49
	ASD-class 1	0	7	4	2	13	o	5	6	1	1	13
	ASD-class *2*	0	6	2	0	8	0	3	1	0	0	4
non Symptomatic		12	6	6	9	33	13	4	6	7	7	37

% of mice Symptomatic	ASD-class 1	0	54	40	18		0	56	50	13	14	
	ASD-class 2	0	46	20	0		0	33	8	0	0	
non Symptomatic		100	46	60	82		100	44	50	55	86	

*χ*^2^ vs. Wt-Wt-CD			4.40E-36	1.50E-09				4.15E-35	2.99E-24			
*χ*^2^ vs. Het-Het-CD					2.20E-09					9.40E-15	1.30E-13	

*Number and percent of non-symptomatic, symptomatic-class 1 and symptomatic-class 2 mice among the experimental groups. Because the small group size of male Het-Het-PN (group 5, *n* = 3) precluded statistical analysis and extraction of the percent of symptomatic mice, the data for these mice were excluded from the table. ASD-Class 1, repetitive; ASD-class 2, repetitive *+* social deficit, Chi Square test with *post hoc* Holm–Bonferroni correction.*

Mice were fed with the rodent food 2018S TEKLAD *ad libitum*. Nutritional enrichment comprised drinking water supplementation with 9 mg/ml folic acid, 2% betaine, and 2% choline.

### *Mthfr* Genotyping

Offspring were genotyped using polymerase chain reaction (PCR) amplification of DNA isolated from toe clips. The three primers used in the PCR analysis were: sense primer 1 (5′-GAA GCA GAG GGA AGG AGG CTT CAG-3′) in exon 3, sense primer 2 (5′-AGC CTG AAG AAC GAG ATC AGC AGC-3′) in the *neo*^r^ gene, and antisense primer 3 (5′-GAC TAG CTG GCT ATC CTC TCA TCC-3′) in intron 3.

### Evaluation of C1 Metabolism

Liquid chromatography-tandem mass spectrometry was employed to measure tissue concentrations of C1 metabolites in the liver, cerebral cortex and basal forebrain. The C1 metabolites, including choline, betaine, methionine, dimethylglycine, acetylcholine, phosphocholine, phosphatidylcholine, glycerophosphocholine, and sphingomyelin were measured according to Koc et al. (2002), with slight modifications based on the instrumentation (Yan et al., 2014). S-adenosylmethionine and S-adenosylhomocysteine were measured by the method of Kim et al. (2005).

### Adult Behavior

At age 3 months, mice were run through a battery of behavioral tests to assess ASD-like behavior. A week before the experiments, the mice were separated and placed in individual cages to avoid any effects of social hierarchy on mouse behavior. During that week, the mice were handled daily by the experimenter for 2 min to acclimatize them to the experimenter’s presence. All experiments were videotaped and analyzed offline using EthoVision software (Noldus, Netherlands). All mice were tested daily between 16:00 and 20:00. In all of the behavioral tests, males and females were tested in separate sessions. The testing arena was cleaned between trials with 70% ethanol.

#### Repetitive Behavior

Two tests were used to address different aspects of repetitive behavior. In the marble burying test, sixteen dark green marbles (15 mm diameter) were arranged in a grid on top of the bedding material (4 cm deep) in a clean cage. Mice were allowed to explore the cage for 10 min, and the cage was photographed for later analysis by two observers blind to group identity. The number of marbles buried (>50% of the marble covered) was recorded (Thomas et al., 2009). In the nest building test. Soft tissue paper was folded in half three times to form an eight-layer object with a final size of 4 cm × 4.5 cm and then placed 2 cm from the wall opposite the water outlet in a clean cage. Nest width, length and height, material processing and nest quality were measured after 2 and 24 h, and the later measurement was used to classify the mice (Lijam et al., 1997). Different features of the nest were used to assay animal welfare and repetitive behavior. While material processing reflects excessive repetitive behavior, nest quality represents the animal’s well-being as described before (Sadigurschi and Golan, 2018).

#### Open Field Task

General behavior: mobility, anxiety, and exploratory behavior were tested in an open field arena. Mice were placed for 5 min in a circular arena 55 cm in diameter with 20-cm high walls. The tested variables were mean walking velocity and percentage of time in the arena during which the mice were in motion, the total durations of time the mice spent in the center and margin of the field, the number of entries into these areas, and rearing frequency (Levav-Rabkin et al., 2010).

#### Social Interaction

Initiation of social interaction was examined in a three-chambered apparatus, in which each chamber measured 40 cm × 20 cm × 22 cm (*length* × *width* × *height*). The apparatus was similar in dimensions and design to one described previously (Nadler et al., 2004; Kezurer et al., 2013). Each chamber of the apparatus (S1, Center, and Empty chambers) was divided from the adjacent chamber(s) by a panel with a hole (2 cm × 3 cm) at its base that could be opened for the mouse to pass through. On the first day of the test, sociability was evaluated. An unfamiliar adult mouse, Stranger 1, was placed in the left-hand chamber in a plastic box while the other, identical box on the right-hand side of the apparatus was empty. Stranger 1 had the same genetic background and gender as the subject mouse and had been habituated to the box before the test. The tested animal was placed in the central compartment with the dividers inserted for a 10-min adaptation period. The dividers were then removed, allowing the test subject to freely explore all three chambers during a 10-min test session. On the following day, preference for social novelty was evaluated. The original stranger mouse (Stranger 1, now a familiar mouse) remained in its box on the left side of the apparatus. A new unfamiliar mouse (Stranger 2) was placed in the previously empty box on the opposite side of the apparatus. Measurements of the number of entries by the tested mouse into the chambers, the total duration of time it spent in each chamber, the time the tested mouse spent sniffing Stranger 1 and Stranger 2 and its movement parameters were taken on both days (Nadler et al., 2004; Crawley, 2007; Kezurer et al., 2013).

### Immunoblot Analysis

Mouse cerebral cortex was homogenized in the presence of protease inhibitors (Sigma-Aldrich) with a hand grinder (15 strokes) in 0.3 ml homogenization buffer (320 mM sucrose, 5 mM Tris-base, pH 7.4). Homogenates were centrifuged for 10 min at 800 × *g*. The resulting supernatant was collected as cellular fraction and used for the analysis of proteins in the glutamate and GABA pathways, and the pellet was processed for its nuclear fraction to assess histone levels and histone modifications. Proteins were separated by using 7.5% sodium dodecyl sulfate-polyacrylamide gel electrophoresis (SDS-PAGE) under reducing conditions. After the proteins were transferred to a nitrocellulose membrane, the membranes were probed with the following primary antibodies: rabbit anti-GluR1 (1:1000, Chemicon International Inc, Temecula, CA, United States), rabbit anti NR2A and rabbit anti NR2B (1:1000 and 1:500, respectively. Novus Biologicals), mouse anti PSD95 and mouse anti-GluR2 (1:50 and 1:100, respectively, NeuroMab), mouse anti β-actin, rabbit anti GAD65/67 and mouse anti KCC2, (1:3000, 1:5000, 1:1000, respectively, Sigma-Aldrich), mouse anti-VGAT and mouse anti-Gephyrin (1:10,000 and 1:5000, respectively, Synaptic Systems, Göttingen, Germany), and monoclonal anti-NKCC1 (T4; 1:2500, developed by Christian Lytle, obtained from the Developmental Studies Hybridoma Bank developed under the auspices of the NICHD and maintained by the University of Iowa, Department of Biological Sciences, Iowa City, IA, United States). Anti-mouse IgG (1:5000, Chemicon) and anti-rabbit IgG horse-radish peroxidase-conjugate secondary antibody (1:2500-1:5000, Upstate, Lake Placid, NY, United States) were used for detection. Visualization was obtained with an enhanced chemiluminescence system (ECL). Quantification was performed with Image Lab 2.0 software (Bio-Rad). The results of each trial were normalized to the trial mean. Two to four independent repeats of each sample were averaged.

### Histone Methylation

Tissue was prepared as described above. Proteins were separated by using 7.5% SDS-PAGE under reducing conditions. After the proteins were transferred to a nitrocellulose membrane, the membranes were probed with either monoclonal mouse anti-dimethylated H3K9 antibody (1:1000, AB1220, Abcam, Austin, TX, United States) or the polyclonal rabbit anti-dimethylated H3K27 (1:1000, ab24684 Abcam, Austin, TX, United States). Secondary anti-mouse IgG horse-radish peroxidase-conjugate secondary antibody (1:5000, Chemicon) or anti-rabbit IgG (1:2500–1:5000, Upstate, Lake Placid, NY, United States) was used for detection.

### Statistical Analysis

Statistical analyses were performed using SPSS 21.0 software. Univariate and multivariate tests with generalized linear models were used to analyze the effects of maternal genotype, offspring genotype, diet and sex on ASD-like behavior, C1 metabolites, brain proteins and H3 methylation and to examine the possible interactions between the independent factors. Odds ratios (ORs) were calculated using the profile likelihood method. Since Wt dams have only Wt offspring and enriched diet was tested only in the Het-Het offspring as described in the study design, some interactions could not be addressed. Therefore, certain tests were only run for some of the groups, as specified in the results section. Samples larger or smaller than the mean ± 1.5 × SD were excluded. To ensure that mouse behavioral classifications would be in line with the diagnostic criteria for ASD in humans, the mouse classification for autistic-like behavior included two subtypes of ASD-like behavior, repetitive behavior and impaired social interaction. Mice were classified as non-symptomatic, symptomatic-Class 1 (presenting repetitive behavior in the marble burying and nest building tests with a value above a threshold of group mean + 1 SD) or symptomatic-Class 2, which included those mice that not only met the criteria for Class 1, but also that had impaired social behavior in the sociability test, as defined by shorter sniffing time in the mouse area vs. sniffing time in the empty area. Differences with *p*-values < 0.05 were regarded as significant. Results are presented as mean ± SEM.

## Results

### ASD-Like Behavior

**FIGURE 1:**
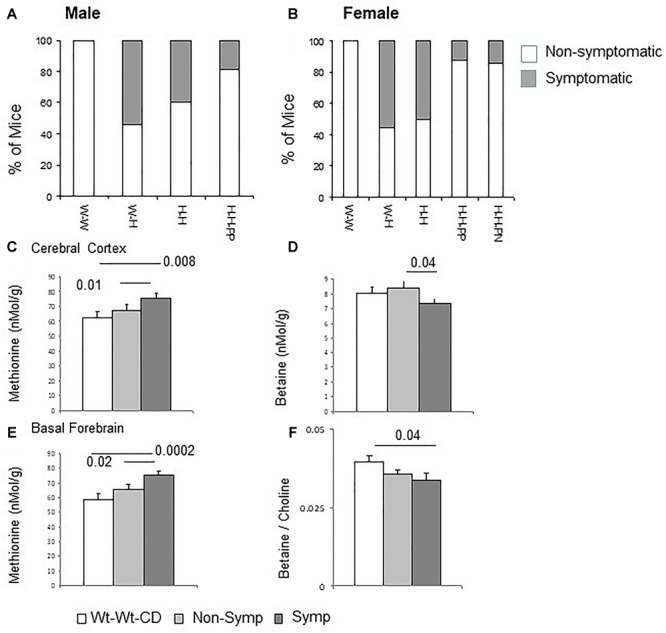
ASD-like behavior – proportion of symptomatic male **(A)** and female **(B)** mice among experimental groups. C1 metabolism levels categorized by phenotype (Male + Female) in the cerebral cortex for methionine **(C)** and betaine **(D)** and in the basal forebrain for methionine **(E)** and for betaine/choline ratio **(F)**. N = Wt-Wt-CD, 28; Non-Symptomatic, 78; Symptomatic, 21. **(A,B)** Wt-Wt-CD (W-W); Wt-Het-CD (W-H); Het-Het-CD (H-H); Het-Het-PP (H-H-PP); Het-Het-PN (H-H-PN). N, as detailed in Table 1. Because the small group size of male Het-Het-PN (group 5, *n* = 3) precluded statistical analysis and extraction of the percent of symptomatic mice, the data for these mice were excluded from the figures. Data are presented as means ± SEM. *P*-value refers to the difference compared to Wt-Wt-CD group.

Our group recently assessed the effects of maternal and offspring heterozygote *Mthfr*-KO genotype on mouse behavior in tasks representing autism core symptoms (Sadigurschi and Golan, 2018). In the current work, mice performances in the tests for assessing repetitive behavior and social approach as well as exploration and anxiety were analyzed for the effects of the independent factors. Detailed results for offspring and maternal genotypes, sex and diet are presented in the Supplementary Material, Behavior Tables (Tables S1–S5). These results were used to characterize each mouse as symptomatic or non-symptomatic according to the ASD-like profile (Figure 1). The frequency of symptomatic mice differed among the groups (*P* < 0.001, chi-square test). Mice in the Wt-Wt-CD group were not symptomatic, whereas a high percentage of symptomatic mice were observed in the offspring of *Mthfr*-deficient dams [Wt-Het-CD and Het-Het-CD groups, 47.6 and 57.1%, respectively (groups 2 and 3)]. *Post hoc* tests indicated a significant decrease in the percentage of symptomatic mice among the Het-Het-PP mice when these were fed only prenatally with an enriched diet (*P* = 0.018 chi-square with Holm–Bonferroni correction). Moreover, feeding mice an enriched diet during only the first postnatal month showed a trend of reduction in the percent of affected mice among the Het-Het group (*P* = 0.07, Holm–Bonferroni correction). Symptomatic mice were further sub-grouped as either Class 1 or Class 2 (Table 1). Among the groups fed enriched diets, the percentage of ASD-Class 1 mice was below 20% for both sexes. The percentage of ASD-Class 2 mice was highest in Wt-Het-CD male mice (46%), and remarkably, the groups fed the enriched diet, either pre or postnatally, contained no ASD-Class 2 mice.

To further evaluate the impact of offspring genotype, sex and diet on behavioral outcome, a logistic regression was run on offspring of *Mthfr*+/- dams (ASD-Class 1 and Class 2 mice were incorporated into a single group) (Supplementary Material, Metabolites Table S1a). ORs were calculated using the profile likelihood method. Among offspring of *Mthfr*+/- dams, PP diet was protective against ASD-like behavior compared to the control diet, with an OR of 0.18 (CI: 0.035, 0.970).

### Effects of Diet and Maternal and Offspring *Mthfr* Genotypes on Biomarkers of C1 Metabolism

*Mthfr* deficiency altered C1 metabolism in the brain tissues of both sexes, with a stronger effect in females than in males. For example, methionine levels in female offspring of *Mthfr*+/- dams were elevated in the cerebral cortex (ANOVA *F*_4,44_ = 3.3, *p* < 0.02), basal forebrain (*F*_4,42_ = 4.5, *p* < 0.005) and liver (*F*_4,44_ = 7.7, *p* < 0.01) (Supplementary Figure S1). Positive associations between the behavioral phenotype and methionine were revealed, showing a significant increase in methionine in the cortex and basal forebrain of symptomatic mice (Class 1 and 2) compared to both the control and the non-symptomatic mice (Figure 1C,E). In addition, lower betaine levels and a lower betaine/choline ratio were found in the cerebral cortex and basal forebrain, respectively, of symptomatic compared to non-symptomatic mice (Figure 1D,E).

#### Cerebral Cortex

Two logistic regression models were performed to assess the effect of the independent variables (offspring genotype, diet and sex) and choline metabolites on ASD outcome among the offspring of *Mthfr*+/- dams. The first model included offspring genotype, diet and sex. The second model included offspring genotype, diet, sex and the choline metabolite concentrations in the cerebral cortex. Metabolites retained in the final model included betaine, choline, glycerophosphocholine, phosphocholine, PtdCho, sphingomyelin, SAM, SAH and SAM:SAH ratio. For the first model, diet was a significant predictor of ASD outcome. For the second model, offspring genotype, SAH, and the SAM/SAH ratio were significant predictors of ASD outcome, while phosphocholine, PtdCho, sphingomyelin and SAM tended (*P* < 0.10) to influence ASD outcome (Supplementary Material, Metabolites Table S1b). In the latter case, the effect of diet (observed in the first model) disappeared, suggesting that choline metabolites may mediate the effect of diet on ASD-like behavior.

#### Basal Forebrain

Two logistic regression models were performed as described in Section “Cerebral Cortex.” The first model included offspring genotype, diet and sex. The second model included offspring genotype, diet, sex and the choline metabolite concentrations in the basal forebrain. Metabolites retained in the final model included betaine, methionine, acetylcholine. For the first model, diet was a significant predictor of ASD outcome. For the second model, methionine tended (*P* < 0.10) to be positively associated with a risk of ASD-like behavior, while betaine tended (*P* < 0.10) to have a negative association (Supplementary Material, Metabolites Table S2). Notably, the effect of diet (observed in the first model) disappeared, suggesting that choline metabolites may mediate the effect of diet on ASD-like behavior.

#### Liver

A logistic regression analysis of the main effects of the independent factors and metabolites found no association between hepatic C1 metabolites and ASD phenotype (data not shown).

Taken together, the results show that alterations in C1 metabolite levels in the brain were sensitive to offspring and maternal *Mthfr* genotypes, with the greatest sensitivity found in the cerebral cortex.

### *Mthfr* and the GABA Pathway in Mouse Cerebral Cortex

To identify which molecular pathways are altered by the Mthfr deficiency, the two main neurotransmitter systems in the cerebral cortex, the GABAergic and glutamatergic systems, were tested as potential mediators of abnormal behavior. The former system is described here and the latter is described in the following section. To explore possible genotype-driven alterations in the GABA pathway, GAD65/67 and VGAT were chosen to represent the GABAergic cells, and gephyrin, the GABA receptor anchoring protein, was selected as a common postsynaptic molecule. In addition, the ionic transporters KCC2 and NKCC1 were selected to represent the key cellular components involved in determining the intracellular chloride concentration and thus, the hyperpolarizing/depolarizing characteristics of the GABA synaptic potential.

**FIGURE 2:**
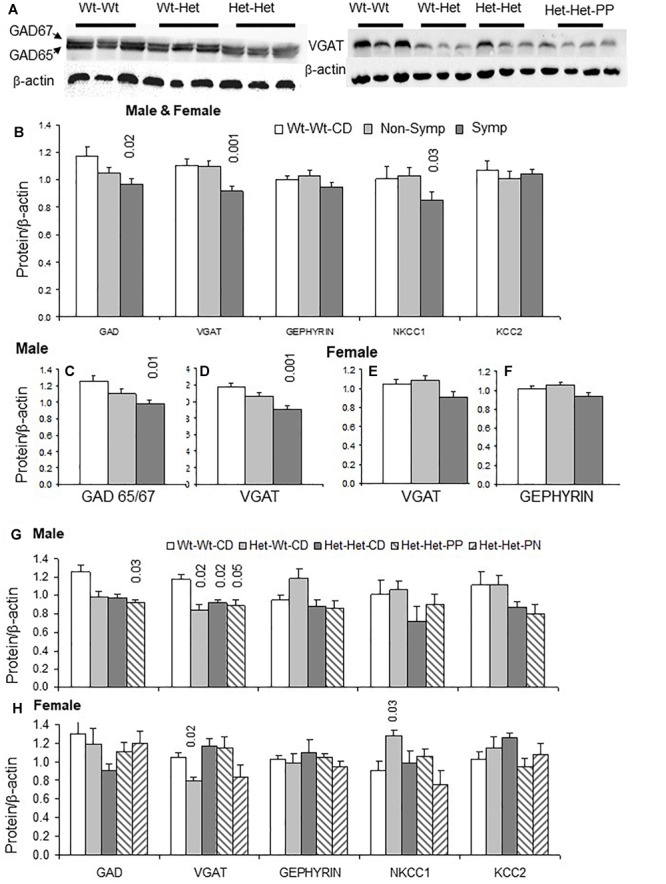
GABA pathway proteins in the cerebral cortex: Effects of maternal and offspring genotypes, diet, sex and behavioral phenotype on protein levels. **(A)** Contains examples of GAD65/67 and VGAT blots. GABA pathway protein levels normalized to beta-actin levels in all mice presented by behavioral categories **(B)** and segregated by sex, showing GAD65/67 and VGAT in males **(C,D)** and VGAT and gephyrin in females **(E,F)**. N, Male, Wt-Wt-CD, 8; Non-Symptomatic, 16; Symptomatic, 8. N, Female, Wt-Wt-CD, 11; Non-Symptomatic, 25; Symptomatic, 9. Analysis by experimental group in males **(G)** and females **(H)**. N, Male, Wt-Wt-CD, 8; Wt-Het-CD, 5; Het-Het-CD, 7; Het-Het-PP, 6. N, Female, Wt-Wt-CD, 9; Wt-Het-CD, 7; Het-Het-CD, 9; Het-Het-PP, 9; Het-Het-PN, 6. *P*-value in the figure refers to the difference obtained by a one-way ANOVA *post hoc* test compared to the Wt-Wt-CD group. Additional values for **(G)**: VGAT Wt-Het-CD vs. Het-Het-CD 0.006; **(B)**: non-symptomatic vs. symptomatic VGAT- 0.003 and NKCC1- 0.04; **(D)**: 0.02; and **(F)**: 0.04. In all other *post hoc* tests, significant differences were found only when compared to the Wt-Wt-CD group, as indicated in the figure. Data are presented as mean ± SEM.

Examples of GAD and VGAT blots are presented in Figure 2A. Cerebral cortex tissue of ASD-like symptomatic mice had significantly lower levels of GAD65/67, VGAT and NKCC1 compared to those in the WT-WT-CD and non-symptomatic mice (Figure 2B). When examining data by sex, significantly reduced levels of GAD65/67 and VGAT were obtained in male mice presenting ASD-like behavior (Figure 2C,D, symptomatic vs. Wt-Wt-CD). Decreased levels of VGAT were also found in ASD-like symptomatic female mice, in whom gephyrin was also reduced compared to non-symptomatic females (Figure 2E,F).

Analyses of all independent factors detected an overall effect of offspring genotype on VGAT (*F*_1,49_ = 5.8, *p* < 0.02) and NKCC1 (*F*_1,49_ = 4.6, *p* < 0.04) and a trend of effect on GAD65/67 levels (*F*_1,4_9 = 3.6, *p* < 0.06). VGAT levels were also sensitive to maternal genotype (*F*_1,46_ = 8.4, *p* < 0.007) (Figure 2). Offspring genotype interacted with sex to modify gephyrin (*F*_1,46_ = 9.2, *p* < 0.005) and KCC2 levels (*F*_1,46_ = 4.0, *p* < 0.05). Gephyrin levels were also sensitive to the maternal genotype × sex interaction (*F*_1,46_ = 5.2, *p* < 0.03). On the other hand, a sex × ASD-like-phenotype interaction was observed for the levels of VGAT (*F*_1,46_ = 4.9, *p* < 0.04), with a stronger effect in male mice (Figure 2G,H).

Analyses of *Mthfr*+/- mice (Het-Het groups) for the effect of nutrition showed that it influenced levels of the main GABAergic system proteins (GAD65/67 *F*_2,26_ = 3.6, *p* < 0.05; VGAT *F*_2,26_ = 3.9, *p* < 0.04; KCC2 *F*_2,26_ = 4.4, *p* < 0.03) and that it had a trend of effect on gephyrin levels (*F*_2,26_ = 3.1, *p* < 0.07). This finding may be explained by the remarkably decreased proportion of mice with ASD-like behavior in the Het-Het groups provided with enriched nutrition. These non-symptomatic mice did not differ from their Wt-Wt-CD counterparts in terms of inhibitory protein levels (Figure 2B).

The genotype-driven alterations in the GABA pathway were also broken down by sex. In males, gephyrin, KCC2 and NKCC1 levels were sensitive to offspring genotype (gephyrin, *F*_1,17_ = 13.7, *p* < 0.002; KCC2 *F*_1,17_ = 4.6, *p* < 0.05; NKCC1 *F*_1,17_ = 5.1, *p* < 0.04), while those of GAD65/67, VGAT and gephyrin were responsive to maternal genotype (GAD65/67 *F*_1,17_ = 7.8, *p* < 0.01; VGAT *F*_1,17_ = 12.7, *p* < 0.003; gephyrin *F*_1,17_ = 6.3, *p* < 0.03) (Figure 2G).

In females, VGAT and NKCC1 levels were altered by the *Mthfr* offspring genotype (*F*_1,18_ = 4.9, *p* < 0.05; *F*_1,18_ = 6.5, *p* < 0.03, respectively), and VGAT was responsive to maternal genotype (*F*_1,18_ = 5.6, *p* < 0.03) (Figure 2G). Mouse behavioral phenotype affected gephyrin (*F*_1,15_ = 6.1, *p* < 0.03) and NKCC1 levels (*F*_1,15_ = 6.0, *p* < 0.03), and it also tended to affect VGAT levels (*F*_1,15_ = 4.4, *p* < 0.06). Nutritional enrichment affected gephyrin (*F*_2,16_ = 4.9, *p* < 0.3) and KCC2 levels (*F*_2,16_ = 7.0, *p* < 0.01) and showed a trend of effect on GAD65/67 levels in *Mthfr*+/- female mice.

As a whole, presynaptic and postsynaptic proteins of the GABA pathway were suppressed by either offspring or maternal genotype or both. Specific associations with the behavioral phenotype were found for several proteins, including NKCC1, a transporter that plays a major role in the regulation of intracellular ion concentrations whose altered functioning can have an impact on the inhibitory nature of GABAergic synaptic potentials (Dzhala et al., 2005). In addition, nutrition had an influence on most of the tested GABA pathway proteins.

### Glutamatergic Synapse Proteins in Mouse Cerebral Cortex

Experience-dependent regulation of the AMPA and NMDA receptor subunits determines synapse properties (Quinlan et al., 1999, 2004; Malinow and Malenka, 2002; Yashiro and Philpot, 2008). The subunits most explored in this context were GluR1 and GluR2 of the AMPAR and NR2A and NR2B of the NMDAR. The localization of these receptors to the membrane and the regulation of their conductance involve molecular interaction with PSD95, a major anchoring protein in glutamatergic synapses.

**FIGURE 3:**
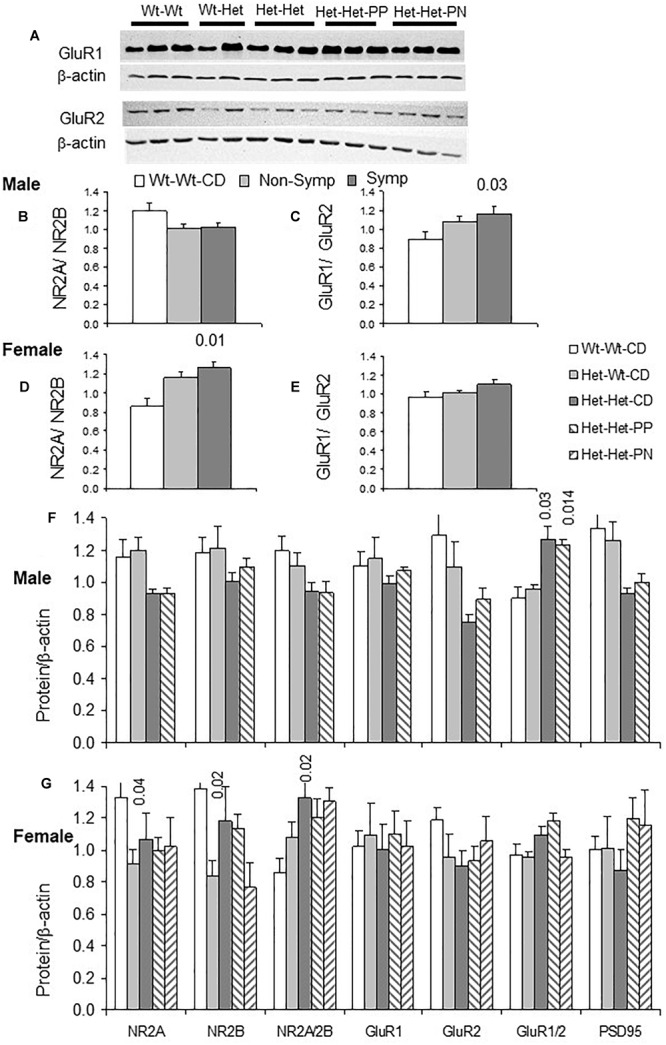
Glutamatergic synapse: Effects of maternal and offspring genotypes, diet, sex and behavioral phenotype on the cerebral cortex. **(A)** Examples of GluR1 and GluR2 blots. GluR1/GluR2 and NR2A/NR2B ratios presented by behavioral categories in males **(B,C)** and females **(D,E)**. N, Male, Wt-Wt-CD, 8; Non-Symptomatic, 16, Symptomatic, 8. Female, Wt-Wt-CD, 11; Non-Symptomatic, 27; Symptomatic, 8. Protein levels of the glutamatergic synapse normalized to beta-actin levels (except where indicated otherwise) in male **(F)** and female **(G)** experimental groups. N, male, Wt-Wt-CD, 8; Wt-Het-CD, 5; Het-Het-CD, 7; Het-Het-PP, 6; female, Wt-Wt-CD, 9; Wt-Het-CD, 7; Het-Het-CD, 9; Het-Het-PP, 9, Het-Het-PN, 6. *P*-value in the figure refers to the difference obtained by a one-way ANOVA *post hoc* test compared to the Wt-Wt-CD group. Additional values are as follows for **(F)**: GluR1/GluR2 Het-Wt-CD vs. Het-Het-CD 0.04, and Het-Wt-CD vs. Het-Het-PP 0.004. In all other *post hoc* tests, significant differences were found only when compared to the Wt-Wt-CD group, as indicated in the figure. Data are presented as mean ± SEM.

Examples of GluR1 and GluR2 blots are shown in Figure 3A. An interaction between sex and behavioral phenotype detailed below is exemplified in Figure 3B–E. Whereas symptomatic males had higher GluR1/GluR2 ratios compared to Wt-Wt-CD males, in symptomatic females, NR2A/NR2B ratios were elevated compared to those in Wt-Wt-CD females.

In line with the large representation of ASD-like behavior among the *Mthfr*-deficient mice, offspring *Mthfr* genotype and sex interacted to alter GluR1/GluR2 (*F*_1,49_ = 5.7, *p* < 0.02) and NR2A/NR2B ratios (*F*_1,49_ = 4.9, *p* < 0.03). In addition, nutrition, sex and behavioral phenotype interacted to modulate GluR1 levels (*F*_1,46_ = 7.2, *p* < 0.01). Among the *Mthfr*+/- population (Het-Het groups), nutrition interacted with the behavioral phenotype to modulate levels of the subunits GluR1 (*F*_2,24_ = 3.9, *p* < 0.04) and GluR2 (*F*_2,24_ = 4.6, *p* < 0.03).

The *Mthfr* genotype in male offspring potentiated the GluR1/GluR2 ratio (*F*_1,11_ = 7.3, *p* < 0.02) and tended to suppress the NR2A/NR2B ratio (*F*_1,16_ = 3.2, *p* < 0.09) (Figure 3F). Although the GluR1 and GluR2 subunits of AMPAR were sensitive to the nutritional enrichment (*F*_1,9_ = 12.9, *p* < 0.016; *F*_1,9_ = 21.2, *p* < 0.006, respectively), this factor had no effect on the NMDAR subunits. In female offspring, maternal genotype differentially suppressed the levels of both the NR2A (*F*_1,29_ = 5.4, *p* < 0.02) and NR2B subunits (*F*_1,29_ = 5.7, *p* < 0.03), resulting in an elevated NR2A/NR2B ratio (Figure 3G) that was not affected by the enriched nutrition. On the other hand, nutritional enrichment interacted with behavioral phenotype in female offspring to alter GluR1 (*F*_1,16_ = 5.4, *p* < 0.02) and GluR2 levels (*F*_1,16_ = 4.4, *p* < 0.04), but in this case, the GluR1/GluR2 ratio was preserved (Figure 3G).

Taken together, the changes observed in this study in the subunit compositions of AMPAR and NMDAR induced by offspring and maternal *Mthfr* genotypes are expected to promote the glutamatergic response to activity and to modify cortical synapse sensitivity to both internal and external inputs.

### *Mthfr* Genotype and Regulation of H3 Methylation

Methyl groups, important products of C1 metabolism, participate in a variety of methylation reactions, among which the methylation of histone proteins has wide-ranging developmental impacts.

An analysis of all mice (male and female) revealed that the dimethylation of histone H3 at lysine 27 (2meH3K27) was susceptible to the interactions between mice genotype and behavioral phenotype, maternal genotype and sex, and behavioral phenotype and sex (*F*_1,44_ = 5.7, *p* < 0.02; *F*_1,44_ = 8.8, *p* < 0.005; *F*_1,44_ = 4.4, *p* < 0.04, respectively), while the genotype × sex interaction showed only a trend of effect (*F*_1,44_ = 3.7, *p* < 0.06). A milder effect was obtained for the dimethylation of histone H3 at lysine 9 (2meH3K9), where trends of interaction were found for sex × genotype × behavioral phenotype (*F*_1,44_ = 2.9, *p* < 0.09), sex × maternal genotype, sex × nutrition, and behavioral phenotype × nutrition (*F*_1,44_ = 3.2, *p* < 0.07; *F*_1,44_ = 3.2, *p* < 0.08; *F*_1,44_ = 3.5, *p* < 0.07, respectively). In addition, offspring genotype altered both 2meH3K27 and 2meH3K9 (*F*_1,44_ = 6.0, *p* < 0.02; *F*_1,44_ = 4.7, *p* < 0.03). Data presented by group are shown in the Supplementary Material H3, Table S1.

**FIGURE 4:**
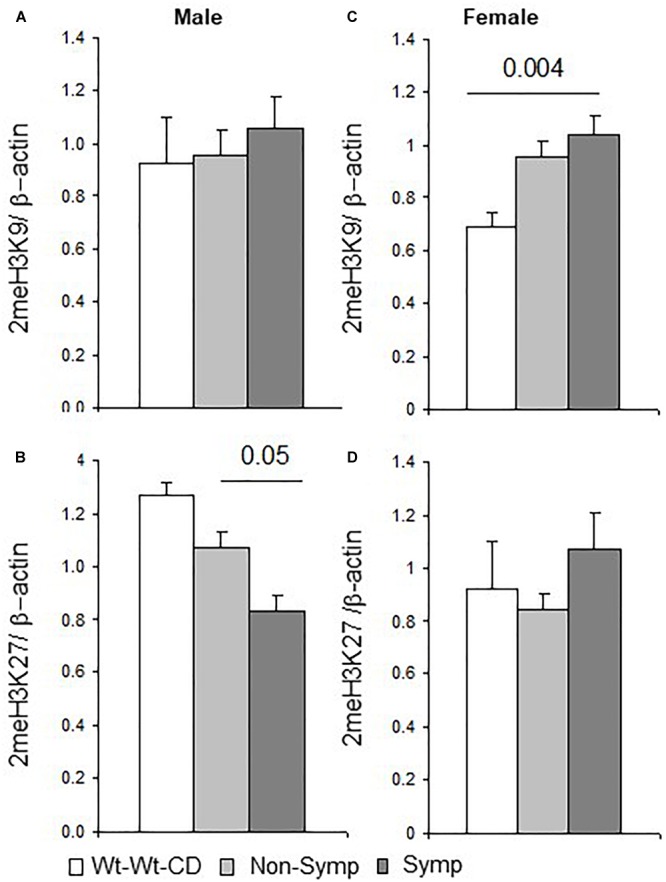
H3 dimethylation – Effects of maternal and offspring genotypes, diet, sex and behavioral phenotype on the cerebral cortex. H3K9 and H3K27 dimethylation levels normalized to beta-actin levels and presented by behavioral categories in males **(A,B)** and females **(C,D)**. N, Male, Wt-Wt-CD, 8; Non-Symptomatic, 16; symptomatic, 8. N, Female, Wt-Wt-CD, 11; Non-Symptomatic, 25; Symptomatic, 8. Data are presented as mean ± SEM. *P*-value refers to the difference between groups connected by a line.

Breaking the analysis down by sex showed that in male mice, as in the whole population, offspring genotype affected 2meH3K9 levels (*F*_1,21_ = 4.8, *p* < 0.04), and maternal genotype showed a trend of effect on 2meH3K27 levels (*F*_1,21_ = 3.3, *p* < 0.08). Reduced levels of 2meH3K27 were obtained in symptomatic compared to non-symptomatic males (Figure 4B). In female mice, an interaction between offspring genotype and the behavioral phenotype led to altered 2meH3K27 levels (*F*_1,32_ = 11.9, *p* < 0.003), which were also significantly altered by each factor alone (genotype *F*_1,32_ = 10.8, *p* < 0.004; behavioral phenotype *F*_1,32_ = 12.3, *p* < 0.003). Maternal genotype also influenced 2meH3K27 levels (*F*_1,32_ = 6.4, *p* < 0.02). In addition, a test of the effect that nutritional enrichment had on MTHFR+/- female mice showed that nutrition and behavioral phenotype interacted to modify 2meH3K9 levels (*F*_1,32_ = 5.0, *p* < 0.05). Indeed, higher 2meH3K9 levels were found in symptomatic but not in non-symptomatic females compared to female mice in the Wt-Wt-CD group (Figure 4C).

In summary, H3 dimethylation on both the K27 and K9 residues was responsive both to the factors tested and to the interaction between these factors. An interaction with sex was also observed in symptomatic mice in the residue sites affected by methylation (Figure 4). This segregation may indicate sex-dependent regulation of gene expression.

## Discussion

A large proportion of the heterozygote *Mthfr+/-* mice showed behavioral impairment characteristic of the ASD-like phenotype that was similar to other rodent models of autism (McFarlane et al., 2008; Bozdagi et al., 2010; Silverman et al., 2011). This finding is in line with epidemiological data indicating that the *MTHFR* 677C > T polymorphism is a risk gene for ASD (Boris et al., 2004; Goin-Kochel et al., 2009; Schmidt et al., 2011, 2012; Guo et al., 2012; Pu et al., 2013). The contribution of the maternal *Mthfr*+/- genotype to the core ASD-like phenotype was recently demonstrated in mice (Sadigurschi and Golan, 2018). Here, we show that a maternal *Mthfr* deficiency was sufficient to increase the proportions of affected offspring. The large percentage of offspring in the Wt-Het-CD group (*Mthfr+/+* mice offspring of *Mthfr*+/- dam) with the ASD-like phenotype emphasizes the critical contribution made by the maternal *Mthfr* genotype to fetal C1 metabolism.

Our data highlight the beneficial effects of early life nutritional supplementation. In particular, our investigation of the core symptoms that segregate symptomatic from non-symptomatic mice elucidated the protective effects of maternal nutritional supplementation (see Figure 1 and Table 1). Supplementation of maternal diet, either prenatal or during the first postnatal month, reduced the proportion of affected offspring, supporting the dependency of fetuses and lactating newborns on an exogenous source of C1 metabolites. Moreover, this observation supports the involvement of C1 metabolism in the autistic phenotype and the feasibility of a diet enriched in C1 nutrients as a means for intervention. This is in line with human studies (Schmidt et al., 2012; Pu et al., 2013; Surén et al., 2013) and with similar observations in the BTBR mouse strain (Langley et al., 2014). From a therapeutic perspective, one of the most intriguing results of our study is the impact of postnatal supplementation. It implies that early diagnosis paired with nutritional intervention adjusted to the child’s genotype/metabolic needs may be a viable, practical means to attenuate autistic manifestations in affected children.

One cannot ignore the possibility that the changes found in this study in the C1 metabolic pathway, either deficiency in or supplementation with metabolites, may have indirect affected outcomes by impacting maternal well-being and maternal care (Francis et al., 1999). This possibility requires further study before it can be definitively excluded.

The potentially deleterious effects of changes in the relative amounts of C1 metabolites have been examined in other studies. The metabolic fingerprint observed in children diagnosed with ASD, regardless of their genotype, is characterized by a reduced ratio of betaine/choline compared to controls (Hamlin et al., 2013); similar relations were observed in the symptomatic vs. non-symptomatic mice in our study (Figure 1) and in the cerebral cortex of offspring of *Mthfr+/-* dams. Thus, betaine to choline ratio may serve in both species as a predictor for ASD. On the other hand, methionine concentration in the mice was associated with both ASD symptomatic behavior and the *Mthfr+/-* genotype (Figure 1), whereas in humans, low levels of methionine were observed in ASD patients (James et al., 2006). The association between the ASD-like behavioral phenotype and an altered cortical SAM/SAH ratio found in our study is in agreement with reports from human studies, in which altered ratios were found in children on the autistic spectrum, regardless of their genotype (James et al., 2006; Vargason et al., 2017). Since SAM is a major methyl donor for methylation reactions, this suggests not only that changes occurred in the DNA methylation capacity, but also that these changes may be related to the modifications observed in the dimethylation of H3 at the K9 and K27 sites. The sex-dependent alterations we observed in H3K9 and H3K27 dimethylation may be involved in the regulation of gene expression associated with the modified proteins and other components, but further study is needed to thoroughly test this possibility.

The possible therapeutic implications of these findings are also supported by the interesting tendency observed in our study for altered cortical levels of the phospholipids PtdCho and sphingomyelin and of the phospholipid precursor phosphocholine in the symptomatic mice. The addition of these choline metabolites to the model removed the effect of diet on ASD-like behavior, suggesting that these choline metabolites may mediate this relationship. In addition to being observed in the whole population, the particularly high sensitivity of these choline metabolites to the interaction between genotype and phenotype in the male offspring suggests another route to modify cortical neuron function. The phospholipid compositions of neuronal membranes are heterogeneous (Prinetti et al., 2000; Yang and Wang, 2009), vary with age (Mateos et al., 2010) and have a significant effect on neuronal function (North and Fleischer, 1983; Fantini and Barrantes, 2009; Colon-Saez and Yakel, 2011; Cornelius et al., 2015). The distributions of PtdCho and sphingomyelin in neuron membranes have been associated with the enrichment of particular ion transporters and receptors in membrane niches (Colon-Saez and Yakel, 2011; Cornelius et al., 2015). Thus, it is possible that in addition to the changes shown above in the concentrations and compositions of synaptic proteins, their differential membrane distributions found in the cortices of mice presenting the ASD-like phenotype, which are mediated by phospholipid composition, also contribute significantly to the phenotype’s incidence.

It was suggested that neuronal networks in autistic brains suffer from a shift in the balance between excitatory and inhibitory processes in the brain (Yizhar et al., 2011; van de Lagemaat et al., 2014; Nelson and Valakh, 2015). Representative proteins of both the GABAergic and glutamatergic pathways tested in this study support impacts on both systems in the symptomatic mice. For example, we found that the ratio of glutamate receptor subunits was modified, such that receptor subunits with higher sensitivities to activity were favored (Flint et al., 1997; Quinlan et al., 1999; Passafaro et al., 2001; Malinow and Malenka, 2002); Relative to mice in the Wt-Wt-CD group, symptomatic males exhibited higher GluR1/GluR2 ratios and symptomatic females had higher NR2A/NR2B ratios. Sex hormones, particularly estradiol, have been shown to have a significant influence on synaptogenesis and synaptic plasticity in the glutamatergic synapse (Segal and Murphy, 2001; Liu et al., 2008).

**FIGURE 5:**
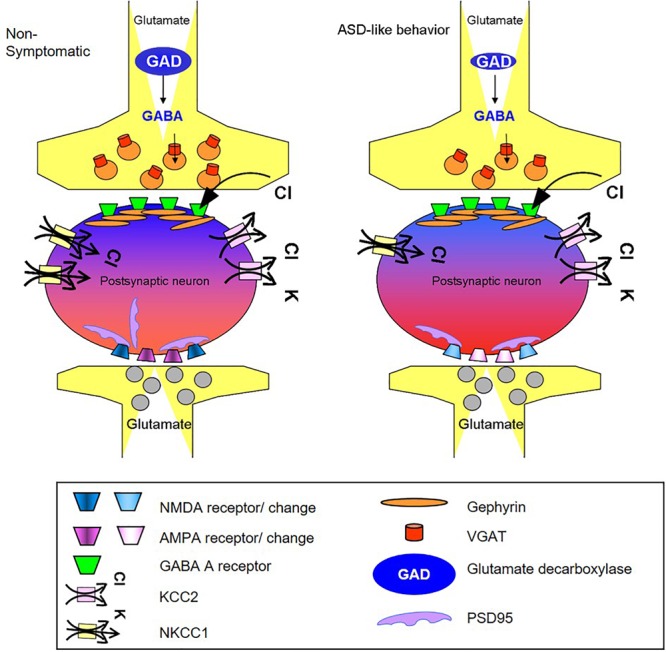
Schematic summary of changes in GABA and glutamate synapse components obtained in the cortex of mice presenting the ASD-like phenotype. The illustration shows the possible molecular origin of the neuronal perturbation that leads to ASD-like behavior in Mthfr deficient mice. The cellular consequences of the changes in GABAergic and glutamatergic proteins observed in the current study: the GABAergic pre-synapse in the ASD-like cortex contains a lower number of vesicular GABA transporters (red) and lower levels of GAD (blue) compared to non-symptomatic cortex. The post-synapse site in the neuron of the ASD-like cortex contains a lower number of NKCC1 transporters (yellow), and thus, GABA receptor (green) activation may result in a less hyperpolarized potential compared to the non- symptomatic cortex (represented by lighter blue color of the neuron in the GABAergic synapse region). The glutamatergic synapse in the cortex of ASD-like mice had a smaller number of PSD-95 molecules (purple). Furthermore, the subunit compositions of the AMPA (purple) and NMDA (blue) receptors in these mice differed from those in the non-symptomatic mice, represented in the figure by the different colors of the receptors. AMPA and NMDA were affected in a sex dependent manner that is not represented in the figure.

Regarding the GABA pathway, its suppression was indicated by decreases in the levels of several key proteins. Taken together, these changes, summarized in Figure 5, are expected to alter cerebral cortex basal activity in symptomatic mice. Furthermore, considering that similar changes take place in other brain regions, the impact on behavior may be even stronger.

Layer specific alterations in the number and distribution of parvalbumin expressing interneurons observed in *Mthfr*-deficient mice (Sadigurschi and Golan, 2018) suggest an involvement of the GABAergic system. Indeed, the *Mthfr*+/- genotype and ASD-like behavior were most robustly associated with the GABAergic pathway in our study. Suppression of presynaptic and postsynaptic proteins in the cortices of symptomatic mice is expected to affect both the GABA input to a variety of cortical neurons and the GABAergic interneuron output. These findings are consistent with evidence linking ASD with impaired GABA function in human research, which comprises genetic (Lionel et al., 2013), expression (Fatemi et al., 2014), histological (Hashemi et al., 2016), functional (Robertson et al., 2016), and intervention studies (Brondino et al., 2016). Impaired GABA pathway functionality was also found in mouse models of ASD (Gogolla et al., 2009) and other neurodevelopmental disorders with pre-existing vulnerability (Romano et al., 2014). The data presented in our study suggest a direct interaction between *Mthfr* deficiency, ASD-like behavior and the GABAergic system. Nutritional intervention in the present study included a cocktail of methyl donors comprising folic acid, betaine and choline. It is possible that one of these supplements alone is sufficient to reduce the rate of symptomatic mice among the Het-Het offspring, as was suggested, in human studies, for prenatal folic acid supplementation (Schmidt et al., 2012; Pu et al., 2013; Surén et al., 2013) and for supplementation with folinic acid of 7-year-old ASD children (Frye et al., 2018). However, the finding that the preferred methyl donor in MTHFR-deficient subjects was choline or betaine (Chew et al., 2011; Yan et al., 2011; Ganz et al., 2016) hints at the possibility that prenatal choline or betaine supplementation may address the metabolic needs of this genetic sub-group better. Further studies may be needed to optimize dosages and to test the effects of combination treatments comprising, for example, the addition of other B vitamins to the nutritional supplements.

Taken together, our findings emphasize the critical role of *in utero* C1 metabolism in developmental trajectories that lead to the presentation of autistic behavior. The most robust impact in this study was that of nutritional intervention, either prenatal or during the early postnatal stage. Aberrations in both the GABAergic and glutamatergic pathways suggest that *Mthfr* deficiency is linked to deleterious alterations in the basal cortical circuit activities in the affected mice. These findings also highlight the potential of C1 metabolism screening in pregnant women as a possible target for genotype-dependent intervention that may reduce the risk of poor neurodevelopmental outcome in the fetus. Nonetheless, the potential of postnatal nutritional intervention adjusted to genotype is also promising, and it should be investigated further in future research.

## Ethics Statement

This study was carried out in accordance with the recommendations of ‘the Israeli Council on Animal Care guidelines.’ The protocol was approved by the animal care and use committee of Ben-Gurion University of the Negev.

## Author Contributions

AO, KF, ST, and RY performed the experiments, statistical analysis, and interpreted the results. HG and MC designed the research. HG drafted the manuscript. AO, ST, and MC contributed to manuscript revision.

## Conflict of Interest Statement

The authors declare that the research was conducted in the absence of any commercial or financial relationships that could be construed as a potential conflict of interest.

## Abbreviations

AMPA: a-amino-3-hydroxy-5-methyl-4-isoxazolepropionic acid
ASD: autism spectrum disorder
CD: control diet
GAD65/67: glutamate-decarboxylase 65 and 67 isoforms
GABA: gamma -aminobutyric acid
GluR1: AMPA receptor subunit 1
GluR2: AMPA receptor subunit 2
Het: MTHFR+/-
KCC2: K^+^-Cl^-^ transporter
Met: methionine
MTHFR: methylene-tetrahydrofolate reductase
NKCC1: Cl^-^ transporters Na^+^-K^+^-2Cl^-^ co-transporter
NMDA: *N*-methyl-di-aspartate
NR2A: NMDA receptor subunit 2A
NR2: NMDA receptor subunit 2B
PKA: protein kinase A
PN: enriched diet during postnatal days 0–30
PP: enriched diet during periconceptional period and pregnancy
PSD95: postsynaptic density protein 95
VGAT: Vesicular GABA transporter
Wt: wild-type
