# Supplementary material for: Prenatal Nutritional Intervention Reduces Autistic-Like Behavior Rates Among Mthfr-Deficient Mice
Source: Front Neurosci. 2019 May 2;13:383. doi: 10.3389/fnins.2019.00383 (PMC6511811; doi:10.3389/fnins.2019.00383)
Supplement: Supplementary file 1 [file Image_1.pdf]

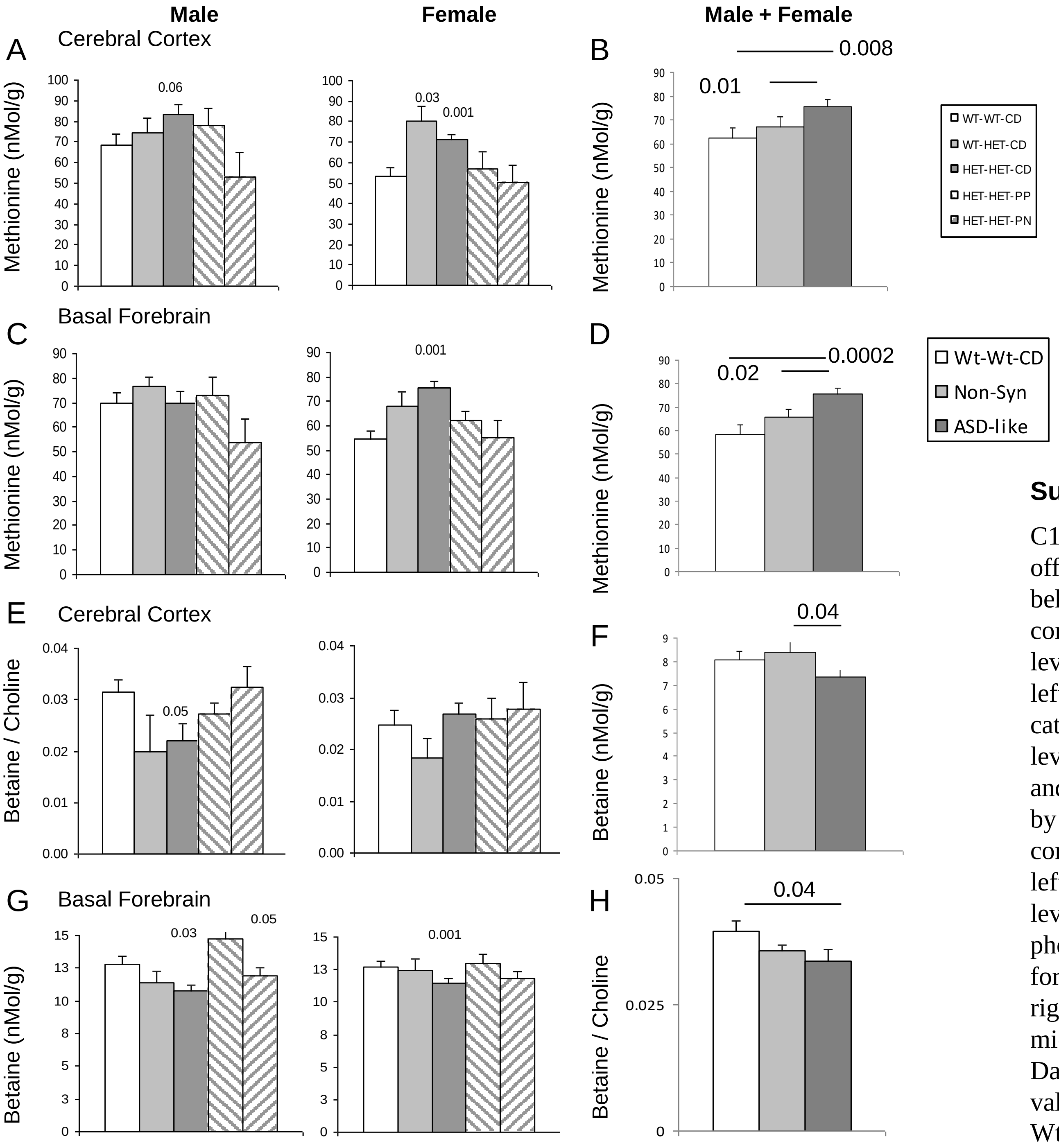

Supplementary figure 1:

C1 metabolism: Effects of maternal and offspring genotypes, diet, sex and behavioral phenotype on the cerebral cortex and the basal forebrain. Methionine levels in the cerebral cortex of male (A, left), female (A, right) and all mice categorized by phenotype (B). Methionine levels in the basal forebrain of male (C left) and female (C right) and all mice divided by behavioral phenotype (D). Cerebral cortex betaine/choline ratio in males (E left) and females (E right) and betaine levels in all mice divided by behavioral phenotype (F). Betaine levels in the basal forebrain of males (G left) and females (G right) and Betaine/Choline ratios in all mice divided by behavioral phenotype (H). Data are presented as mean +/- SEM. P value refers to the difference compared to Wt-Wt-CD group.
